# Intravascular Lithotripsy for Suprarenal Coral Reef Aorta: A Novel Endovascular Approach to Complex Calcified Disease

**DOI:** 10.7759/cureus.99086

**Published:** 2025-12-12

**Authors:** Thalis Charalambous, Fotios Sousamlis, Grigorios Katsantouris, Eleftherios Gerardos, Nikolaos Zannes

**Affiliations:** 1 Radiology Department, Sismanogleio General Hospital, Athens, GRC

**Keywords:** calcified aortic stenosis, coral reef aorta, endovascular therapy, intravascular lithotripsy, visceral artery stenosis

## Abstract

Coral reef aorta (CRA) is a rare atherosclerotic disease characterized by the presence of exophytic, calcified lesions protruding into the aortic lumen, causing severe stenosis of the aorta and its visceral and renal branches. Clinical presentation varies depending on the location and extent of the lesion. Treatment options include open surgery and endovascular approaches. Intravascular lithotripsy (IVL) is a novel endovascular technique that delivers acoustic shockwaves to modify calcified lesions, allowing luminal expansion. We report the case of a 76-year-old female with severe stenosis of the suprarenal aorta, right renal artery, superior and inferior mesenteric artery, and occluded left renal artery. Due to the anatomical complexity and lack of suitable landing zones, the suprarenal aorta was treated with IVL alone without stent or stent-graft placement. To preserve mesenteric perfusion, a balloon-expandable stent was deployed in the inferior mesenteric artery, which served as the dominant supply due to severe superior mesenteric artery stenosis. Following IVL, the patient reported immediate symptom resolution with no periprocedural complications. This case highlights the potential role of IVL as a safe, effective standalone treatment for complex CRA cases.

## Introduction

Coral reef aorta (CRA), first described by Qvarfordt et al. [1], is a rare and severe form of atherosclerosis, characterized by dense, exophytic calcified plaques that protrude into the aortic lumen. These lesions typically involve the juxtarenal and visceral segments of the aorta. They may extend into the iliac arteries and lead to significant narrowing and subsequent visceral and peripheral ischemia, resulting in life-threatening complications. To date, fewer than 130 cases have been reported, underlining both the rarity and probable underdiagnosis of the condition [2]. Clinical presentation varies depending on the distribution of calcification and may include renal dysfunction, intermittent claudication, refractory hypertension, or intestinal angina [2,3].

Open repair, including aortic endarterectomy or bypass, has been the standard treatment [4], but it is associated with significant perioperative morbidity. Endovascular repair offers a less invasive alternative but is often limited by the rigidity of heavily calcified lesions, which can increase the risk of arterial dissection, perforation, or rupture during high-pressure balloon angioplasty [5]. Intravascular lithotripsy (IVL) is a novel endovascular technique that uses acoustic shockwaves to modify heavily calcified plaques, enabling vessel dilatation at low balloon pressures. While IVL is increasingly used in coronary and peripheral interventions, its use in CRA remains limited to a few case reports and case series [6-12].

This study reports the case of a 76-year-old female who presented with intestinal angina and intermittent claudication due to extensive suprarenal, visceral, and renal calcifications consistent with CRA. Because no adequate landing zones were available, the suprarenal aortic lesion was treated with IVL alone, without stent-graft placement. The inferior mesenteric artery, which was the dominant splanchnic supply due to severe superior mesenteric artery stenosis, was selectively revascularized and stented to preserve mesenteric perfusion. This case contributes to the limited evidence supporting IVL as a potential therapeutic option in anatomically complex CRA.

## Case presentation

**Figure 1:**
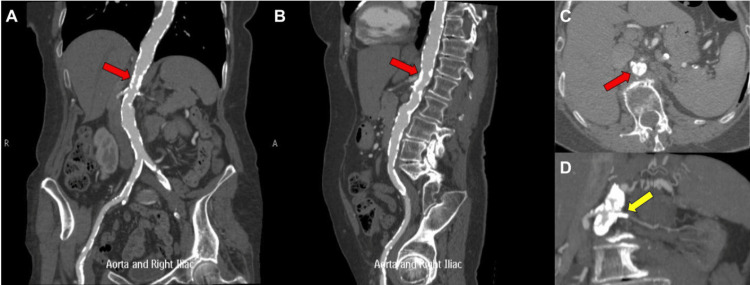
CT angiography of the abdominal aorta (A) Coronal, (B) sagittal, and (C) transverse computed tomography angiography images demonstrate prominent suprarenal intraluminal calcifications measuring approximately 3 mm (red arrow). (D) Coronal computed tomography angiography image shows complete occlusion of the left renal artery (yellow arrow). CT: computed tomography

A 76-year-old female, with hypertension, chronic kidney disease, and dyslipidemia, was referred for evaluation of postprandial abdominal pain and severe lifestyle-limiting bilateral claudication. Computed tomography angiogram of the abdomen and pelvis demonstrated extensive calcifications involving the suprarenal aorta, with the narrowest luminal diameter measuring ~8 mm, located approximately 8.5 mm above the origin of the right renal artery (Figure 1A-1C). Severe stenosis of the right renal artery and complete occlusion of the left renal artery were noted, the latter evidenced by the absent nephrogram (Figure 1D). Additionally, severe stenoses were identified at the origins of both the superior and inferior mesenteric arteries. Because of the extent of calcification and absence of suitable landing zones, the patient was considered unsuitable for endovascular repair. The patient, therefore, underwent percutaneous revascularization with IVL.

**Figure 2:**
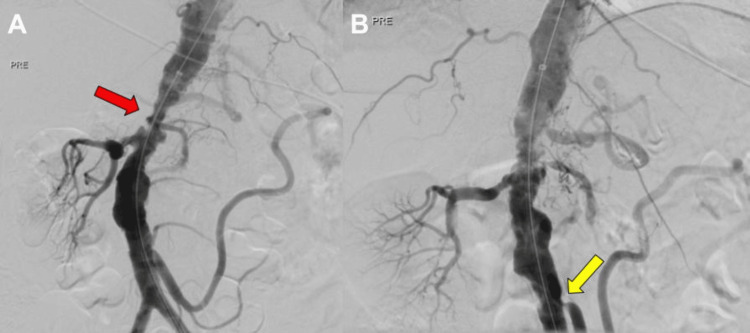
Digital subtraction angiography of the abdominal aorta (A) Lateral and (B) anterior-posterior view demonstrating severe narrowing of the suprarenal aorta (red arrow), absent left nephrogram, and narrowing of the origin of the inferior mesenteric artery (yellow arrow).

**Figure 3:**
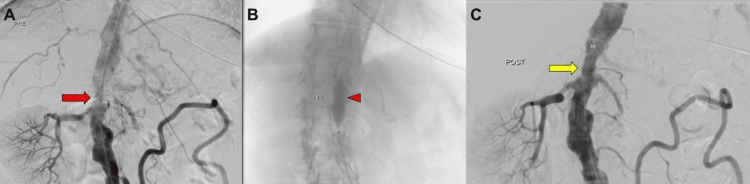
Intraoperative digital subtraction angiography during IVL treatment of the suprarenal aorta (A) Digital subtraction angiography shows significant stenosis of the suprarenal aorta (red arrow). (B) A 10 x 30 mm IVL Shockwave balloon L6 was positioned in the suprarenal aorta (red arrowhead). (C) Post-IVL digital subtraction angiography demonstrates satisfactory dilatation of the treated segment of the suprarenal aorta (yellow arrow). IVL: intravascular lithotripsy

Digital subtraction angiography via left common femoral artery access confirmed the computed tomography angiography findings (Figure 2, Figure 3A). Following diagnostic angiography, a 45 cm, 8F Flexor Check-Flo introducer sheath (Cook Medical, Bloomington, IN, USA) was advanced into the aorta. Predilation of the lesion was performed using a 4 × 20 mm Viatrac balloon catheter over a 0.014-inch ES guidewire to facilitate device passage. IVL was subsequently performed, delivering 240 pulses using a 10 × 30 mm Shockwave L6 balloon inflated to 4 atm, followed by a 12 × 30 mm IVL balloon inflated to 6 atm to further modify the heavily calcified lesions (Figure 3B). Following IVL, angiography confirmed satisfactory dilation of the suprarenal aortic segment (Figure 3C).

**Figure 4:**
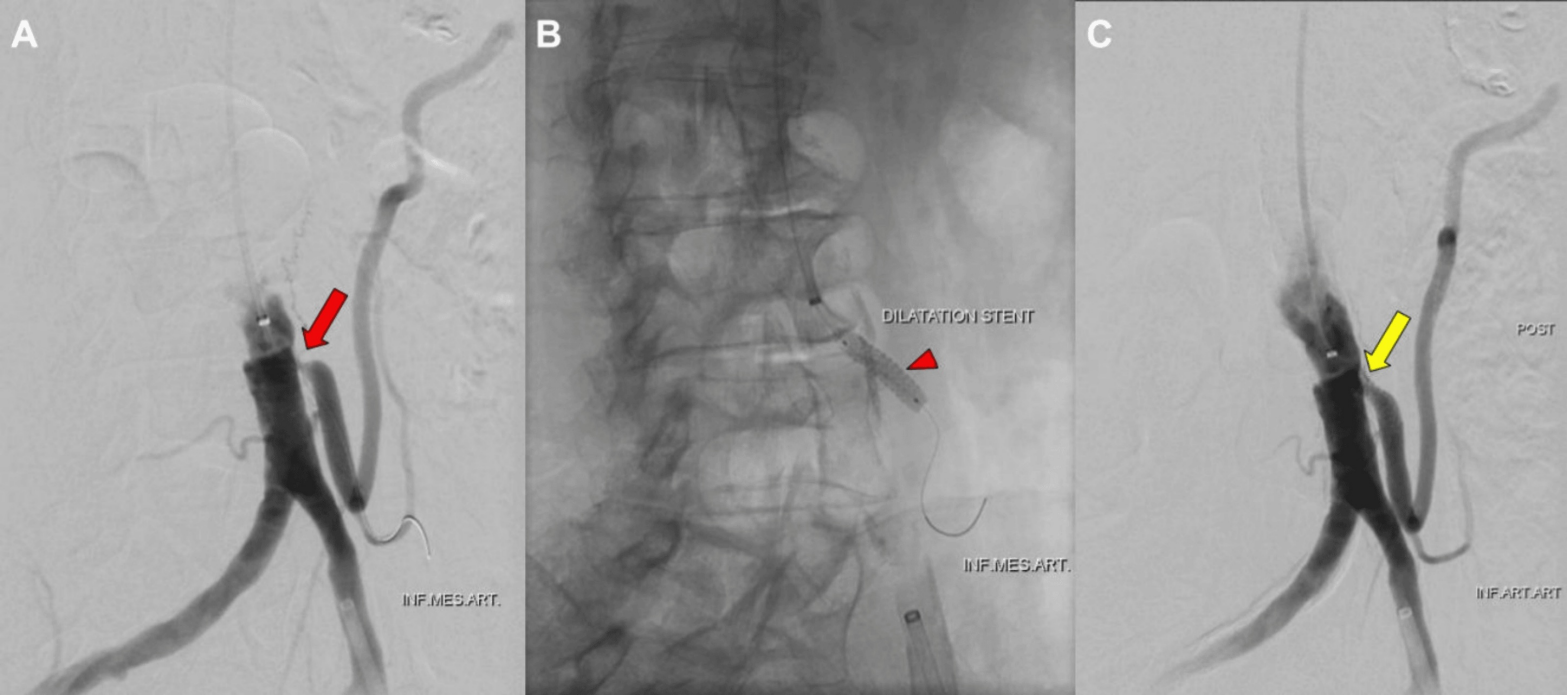
Intraoperative digital subtraction angiography of inferior mesenteric artery revascularization (A) Intraoperative digital subtraction angiography showing significant stenosis of the origin of the inferior mesenteric artery (red arrow). (B) Placement of a balloon expandable stent following dilatation with a drug eluting balloon (red arrowhead). (C) Digital subtraction angiography showing satisfactory dilatation of the origin of the inferior mesenteric artery (yellow arrow).

Additional access was obtained via the left brachial artery to enable targeted revascularization of the inferior mesenteric artery, which served as the dominant mesenteric supply in the setting of critical superior mesenteric artery stenosis. Revascularization was performed using a 7 × 40 mm drug-eluting balloon, followed by an 8 × 40 mm drug-eluting balloon (Cardionovum, Bonn, Germany). To maintain IMA patency, we used a 5.5 mm × 18 mm balloon-expandable stent (Abbott Vascular, Santa Clara, CA, USA) (Figure 4B). Final angiography demonstrated adequate dilation of the IMA origin (Figure 4C). The patient experienced immediate symptom relief without periprocedural complications.

## Discussion

CRA is a rare and severe manifestation of atherosclerotic disease characterized by calcified, exophytic plaques that protrude into the aortic lumen, leading to significant stenosis of visceral and renal vessels. Although its precise pathophysiology remains unclear, it is hypothesized that dysregulation of calcification inhibitors, such as fetuin-A and uncarboxylated Matrix Gla Protein, is implicated in the development of extreme ectopic calcification, leading to CRA development [13]. CRA has an estimated prevalence of 0.6% in the population, with fewer than 130 cases reported, reflecting both its rarity and the likelihood of underdiagnosis. CRA typically presents in patients around 50 years of age, significantly younger than those affected by more common forms of occlusive diseases, with risk factors including diabetes mellitus, smoking, dyslipidemia, and hypertension [3]. In our case, although our patient did not have a history of diabetes or smoking, she had notable cardiovascular risk factors, including dyslipidemia and poorly controlled hypertension, which likely contributed to disease development.

Open surgical repair, including aortic endarterectomy and extra-anatomic bypass, has been considered the standard treatment for CRA. However, reported outcomes demonstrate considerable perioperative burden, including more extended hospital stays, frequent complications, repeat interventions, and increased all-cause mortality. By contrast, endovascular techniques, including angioplasty with either balloon or self-expanding stents, have been associated with lower peri- and postoperative complications (2). Nonetheless, in the setting of dense, heavily calcified lesions, adequate luminal dilation requires high-pressure balloon angioplasty, which carries a significant risk of arterial dissection, perforation, or vessel rupture [14].

IVL has recently emerged as a promising modality for plaque modification in heavily calcified vascular lesions. IVL delivers acoustic shockwaves to modify and fracture calcified plaques, achieving dilatation with low pressure and therefore minimizing the risk of vessel injury. These characteristics make IVL particularly suitable for the rigid, exophytic calcifications characteristic of CRA [14]. To date, IVL has been primarily applied in coronary and peripheral interventions [6,15], with only limited reports describing its use in the aorta, and specifically in CRA. Endovascular atherectomy represents another potential strategy for revascularization of peripheral arteries, with results similar to IVL in the treatment of common femoral arteries [16]; however, its role in CRA remains to be studied.

Recent studies describe IVL for CRA in conjunction with covered stents, which are thought to increase long-term patency and reduce the risk of distal thromboembolic events [6,12]. However, the successful deployment of covered stents requires adequate landing zones to ensure stability and maintain perfusion to vital branches, particularly the renal arteries. In our case, the absence of a sufficient landing zone, combined with pre-existing occlusion of the left renal artery, precluded the safe use of a covered stent. Therefore, IVL alone was chosen as the most appropriate therapeutic strategy. A similar approach was recently used [7,11], further supporting the feasibility of IVL treatment alone in anatomically challenging cases without the need for covered grafts or multiple chimney grafts.

Evidence from peripheral vascular interventions further supports the safety and efficacy of IVL. A meta-analysis of nine studies involving 681 patients and 769 lesions demonstrated a mean stenosis reduction of 59.3%, with flow-limiting dissections occurring in only 1.25% of cases and no cases of distal embolization following IVL for peripheral artery disease [17]. These favorable outcomes are attributed to IVL’s low-pressure balloon inflation and localized shockwave delivery, which minimize barotrauma and embolic risk. These findings align with our patient's uncomplicated periprocedural course and immediate symptom resolution.

Several considerations for future practice should be noted. Intravascular ultrasound (IVUS) may offer superior characterization of plaque morphology, more accurate assessment of lesion severity, and improved evaluation of procedural outcomes [18]. However, its clinical use in aortic interventions remains limited due to increased procedural time and cost, and further studies are needed to clarify the added clinical value of IVUS in this context [19]. Although the application of IVL in the management of CRA is increasingly reported, current evidence remains limited to case reports and small case series. Prospective studies with larger cohorts and longer follow-up are required to establish the long-term efficacy, safety, and optimal role of IVL in the management of this rare but clinically significant disease.

## Conclusions

We report the case of a 76-year-old female with severe stenosis of the juxtarenal aorta, right renal artery, superior and inferior mesenteric artery, and occluded left renal artery that was treated with IVL alone. This case contributes to the limited but growing body of evidence suggesting that IVL may be a safe and effective option for endovascular treatment of juxtarenal CRA where stenting is not feasible. Further prospective studies and long-term follow-up data are required to better define the durability, safety profile, and optimal role of IVL in the management of this rare but clinically significant disease.
